# Evaluating the pathological and clinical implications of errors made by an artificial intelligence colon biopsy screening tool

**DOI:** 10.1136/bmjgast-2024-001649

**Published:** 2025-01-06

**Authors:** Harriet Evans, Naveen Sivakumar, Shivam Bhanderi, Simon Graham, David Snead, Abhilasha Patel, Andrew Robinson

**Affiliations:** 1 University of Warwick, Coventry, UK; 2 Histopathology, University Hospitals Coventry and Warwickshire NHS Trust, Coventry, UK; 3 Department of Colorectal and General Surgery, George Eliot Hospital NHS Trust, Nuneaton, England, UK; 4 Department of Colorectal and General Surgery, University Hospitals Coventry and Warwickshire NHS Trust, Coventry, UK; 5 Histofy, Coventry, UK; 6 Department of Computer Science, University of Warwick, Coventry, UK

**Keywords:** COLORECTAL PATHOLOGY, COMPUTERISED IMAGE ANALYSIS, COLORECTAL DISEASES

## Abstract

**Objective:**

Artificial intelligence (AI) tools for histological diagnosis offer great potential to healthcare, yet failure to understand their clinical context is delaying adoption. IGUANA (Interpretable Gland-Graphs using a Neural Aggregator) is an AI algorithm that can effectively classify colonic biopsies into normal versus abnormal categories, designed to automatically report normal cases. We performed a retrospective pathological and clinical review of the errors made by IGUANA.

**Methods:**

False negative (FN) errors were the primary focus due to the greatest propensity for harm. Pathological evaluation involved assessment of whole slide image (WSI) quality, precise diagnoses for each missed entity and identification of factors impeding diagnosis. Clinical evaluation scored the impact of each error on the patient and detailed the type of impact in terms of missed diagnosis, investigations or treatment.

**Results:**

Across 5054 WSIs from 2080 UK National Health Service patients there were 220 FN errors across 164 cases (4.4% of WSI, 7.9% of cases). Diagnostic errors varied from cases of adenocarcinoma to mild inflammation. 88.4% of FN errors would have no impact on patient care, with only one error causing major patient harm. Factors that protected against harm included biopsies being low-risk polyps or diagnostic features were detected in other biopsies.

**Conclusion:**

Most FN errors would not result in patient harm, suggesting that even with a 7.9% case-level error rate, this AI tool might be more suitable for adoption than statistics portray. Consideration of the clinical context of AI tool errors is essential to facilitate safe implementation.

**WHAT IS ALREADY KNOWN ON THIS TOPIC**

- Artificial intelligence (AI) tools are being developed across healthcare, such as IGUANA (Interpretable Gland-Graphs using a Neural Aggregator) which classifies colonic biopsies, aiming to automatically report normal cases and reduce reporting times.
- There is no guidance on how to objectively evaluate AI tools in terms of clinically relevant metrics, with statistical metrics not enough to show how implementation would impact patient care.

**WHAT THIS STUDY ADDS**

- This study shows how a comprehensive pathological and clinical assessment of errors made by an AI tool can be conducted by a multidisciplinary team.
- It highlights reasons for the errors from a pathological stance, which aids algorithm training and improves safety. It shows that only a small percentage of errors actually result in patient harm and details protective clinical factors, showing how important knowledge of the clinical pathway is.

**HOW THIS STUDY MIGHT AFFECT RESEARCH, PRACTICE OR POLICY**

- Clinically focused assessment of AI tools by multidisciplinary teams, including how errors would impact patient care, should be part of research studies and guidelines for AI tool implementation. This would give a more accurate representation of the tool in practice which could facilitate uptake, bringing all the benefits of AI tools into practice.

## Introduction

 The use of artificial intelligence (AI) in medicine has the potential to transform the delivery of healthcare worldwide, improving diagnostics and clinical care and enhancing healthcare efficiency.^1^ In order for these benefits to be realised, both the effectiveness and safety of any tool need to be robustly assessed.^2 3^

For an AI tool to be implemented in practice, it must provide some benefit to patient care, most often through increased diagnostic accuracy, speed and efficiency or improved stratification of care. However, no AI tool will be perfectly accurate, and so errors will inherently be introduced alongside the benefits, potentially resulting in patient harm. This has been identified as one of the most significant disadvantages of AI in healthcare.^4^

Despite the need to understand both the benefits and risks of using an AI tool from a clinical perspective, there is no guidance on how to objectively evaluate AI tools in terms of clinically relevant metrics.^5 6^ Most studies on AI tools are retrospective in nature and use filtered datasets so are not representative of the true clinical scenario.^3 7^ Furthermore, the results of AI algorithms are typically presented in statistical terms, with no consideration of the potential impact of the errors on patient care.^8^ Although classical performance metrics used in AI tool assessment are important in initial tool evaluation, they are not sufficient to understand the impact on patients in real-world use.^5 8^ It is noted that there is often a failure to translate the impact of the technical properties in an AI system into their impact on patient harm and safety of diagnoses.^9^ A recent large meta-analysis of AI tools in digital pathology described promising results with high diagnostic accuracy reported, but data on error rates and their consequences was largely missing.^10^ This is likely to contribute to the so-called ‘chasm’ between development and use, whereby AI tools with great potential are not adopted in the clinical setting.^3^

### The IGUANA algorithm

Histopathology is one of the areas of greatest progress for AI tools in medicine, with the shift to digital pathology facilitating the formation of large data sets of whole slide images (WSIs) to develop AI diagnostic tools.

IGUANA (Interpretable Gland-Graphs using a Neural Aggregator) is an AI algorithm that examines colon and rectal biopsy H&E WSIs and classifies them into normal or abnormal categories.^11^ IGUANA models each WSI of colonic tissue as a graph with interconnected nodes representing each gland and detects 25 features that evaluate gland architecture and nuclear morphology, as well as the arrangement of the glands within the tissue and the inter-gland cell density.^11^

The promising results of IGUANA have recently been published in Gut.^11^ It was tested and internally validated on a cohort of National Health Service (NHS) cases from University Hospitals Coventry and Warwickshire NHS Trust (UHCW) (consisting of 5054 WSIs from 2080 patients) and then tested on three external data sets (with a total of 1537 WSIs from 1211 patients). IGUANA’s predictions were compared with pathologist slide level diagnoses. IGUANA achieved an area under the curve-receiver operating characteristic (AUC-ROC) of 0.98 (SD=0.004) on the UHCW data and a mean AUC-ROC=0.97 (SD=0.007) across the three external data sets. This shows it can successfully categorise slides into normal versus abnormal groups.^11^

The tool is intended to be used in a screening capacity, detecting normal slides that could have an automated report issued without pathologist review, thus reducing the clinical workload on overstretched pathology departments and reducing turnaround times across cases. As a screening tool, the need to achieve high sensitivity is paramount, and the algorithm has shown a high specificity at high sensitivity cut-off values.^11^

Despite these promising statistical results, which suggest it could be suitable as a screening tool and thus invaluable for pathology departments, errors were made by IGUANA. A comprehensive assessment of these errors and their potential impact on patients is greatly warranted.

In this paper we report a detailed pathological and clinical analysis of the errors made by the IGUANA colon biopsy screening tool. This retrospective analysis contributes to a greater understanding of the tool, and how its safety can be improved, thereby facilitating its implementation. This provides an example for clinicians and developers of how to comprehensively consider the impact of AI tool errors, and details the benefits and challenges of such an approach to support wider clinical evaluation of AI tools.

## Methods

This retrospective analysis was performed on the UHCW data set, consisting of 5054 WSIs from 2080 patients sequentially selected from UHCW archives. In this data set 42% of WSIs were abnormal, including a range of commonly seen inflammatory and neoplastic conditions (including inflammatory bowel disease, non-specific inflammation, microscopic colitis tubular adenomas, hyperplastic polyps and adenocarcinoma^11^). The classification of each WSI by IGUANA and the study pathologist was obtained and cross-referenced to calculate the false negative (FN) and false positive (FP) errors. This initially found 230 FN WSIs (4.6%) and 116 FP WSIs (2.3%).

In this study FN errors were considered the most significant errors because, in the instance of automated normal biopsy reporting, specimens containing an unrecognised histopathological abnormality would not be reviewed by a pathologist, and hence not reported to the clinician. This is a high-risk situation where a patient may not receive further required follow-up, investigation or treatment, potentially leading to patient morbidity or even mortality. In contrast, FP errors would result in a normal biopsy being unnecessarily reviewed by a pathologist, but the patient would still ultimately receive the correct diagnosis.

Initial data review excluded 10 FN WSIs because they were either not colonic biopsies (terminal ileal biopsies) and therefore out of scope for IGUANA, or were actually normal slides that were inappropriately coded as abnormal in the study, thus resulting in 220 FN (4.4% of all WSI, 10.4% of abnormal WSI). At a patient level, 7.9% of patient cases had one or more FN errors.

### Pathological assessment

Each FN WSI was independently reviewed by a consultant histopathologist specialising in gastrointestinal pathology and a trainee histopathologist. The reviewer was not blinded to the original diagnosis.

Assessment of each WSI covered a quality assessment of the slide (good/adequate/poor), assessment of the histological features present and assignment of a specific diagnosis and consideration of any factors that would make assessment of the slide difficult. When formulating the diagnosis, if both pathologists agreed on the given diagnosis there was no further review, but WSIs with a discrepancy were re-reviewed to give a final consensus diagnosis.

### Clinical assessment

Pathological information regarding the FN error, and all other specimens in the case were provided, and the patient history, endoscopic findings and clinical records were reviewed by the clinical team. For each FN error the potential level of patient harm that would have ensued was assessed. A scoring system previously used by the Royal College of Pathologists when conducting a duty of care review following a pathologist error was used:^12^

1. No impact on care.
2. Minimal harm (delay in diagnosis or therapy<3 months, or unnecessary non-invasive investigations, or unnecessary therapy without morbidity).
3. Minor harm (delay in diagnosis or therapy>3 months, or unnecessary invasive investigations or unnecessary therapy with minor morbidity).
4. Moderate harm (any moderate morbidity due to delay in diagnosis or treatment, or unnecessary investigations or interventions).
5. Major harm (loss of limb/organ/function of an organ system, or severe morbidity due to delayed or unnecessary therapeutic interventions, or mortality).^12^

The type of clinical impact on the patient was then assessed as to whether the FN would have caused: Delayed diagnosis, Delayed treatment, Delayed surveillance, or No impact. If a benign lesion such as hyperplastic polyp or lipoma was missed, this was graded as no impact as it does not require treatment or surveillance. Similarly, if there was inflammation present on the biopsy but this was not identified by IGUANA, no impact was recorded if the patient was already on treatment (eg, based on endoscopic findings) and this finding made no change. If this was a missed new diagnosis and no treatment was started at the time of endoscopy, then it was recorded as a delayed diagnosis. If the patient was known to have inflammation but a positive biopsy finding would have led to a change in the treatment, this was coded as delayed treatment. In patients where an adenoma was misreported, if this finding made no difference to subsequent surveillance as per the British Society of Gastroenterology guidelines,^13^ this was coded as having no impact.

In one case there was no patient information available on our hospital system so this case was excluded from the analysis, giving 163 cases (from 163 patients) with 219 WSIs for review.

There were two types of clinical evaluation undertaken:

1. If all the colonic specimens taken during that particular colonoscopy were evaluated by the IGUANA tool, harm was recorded at the level of the patient rather than based on the individual biopsy result. For example, if there were six biopsies taken and IGUANA called one negative, if the other biopsies were correctly identified as being abnormal, there was no patient harm as the diagnosis would have been established based on the other biopsy results. If the FN biopsy was the only one where there was inflammation, there was minor (or moderate) harm and a delay in diagnosis or treatment. Patient-level analysis was possible in 129 cases (177 WSIs).
2. In some patients, only some of the colonic biopsies were analysed by the IGUANA tool so the level of harm could not be assessed at the patient level. In these patients, harm was assessed based on the single FN WSI results, as if there were no other biopsies undertaken. For example, if the FN biopsy is misreported as normal when there is inflammation present, harm is considered to be mild (or moderate) even if there were other biopsies that show inflammation as the IGUANA tool has not assessed these so one cannot assume it would identify inflammation. In this analysis, the level of harm is likely to be exaggerated as there may have been other biopsies that could have identified pathology that cannot be included in the assessment of harm. All WSI underwent this type of hypothetical analysis.

### Patient and public involvement

Lay members were involved in the primary IGUANA study to ensure patients are part of the safe development of AI tools but were not involved in this clinicopathological correlation of errors.

## Results

**Figure 1:**
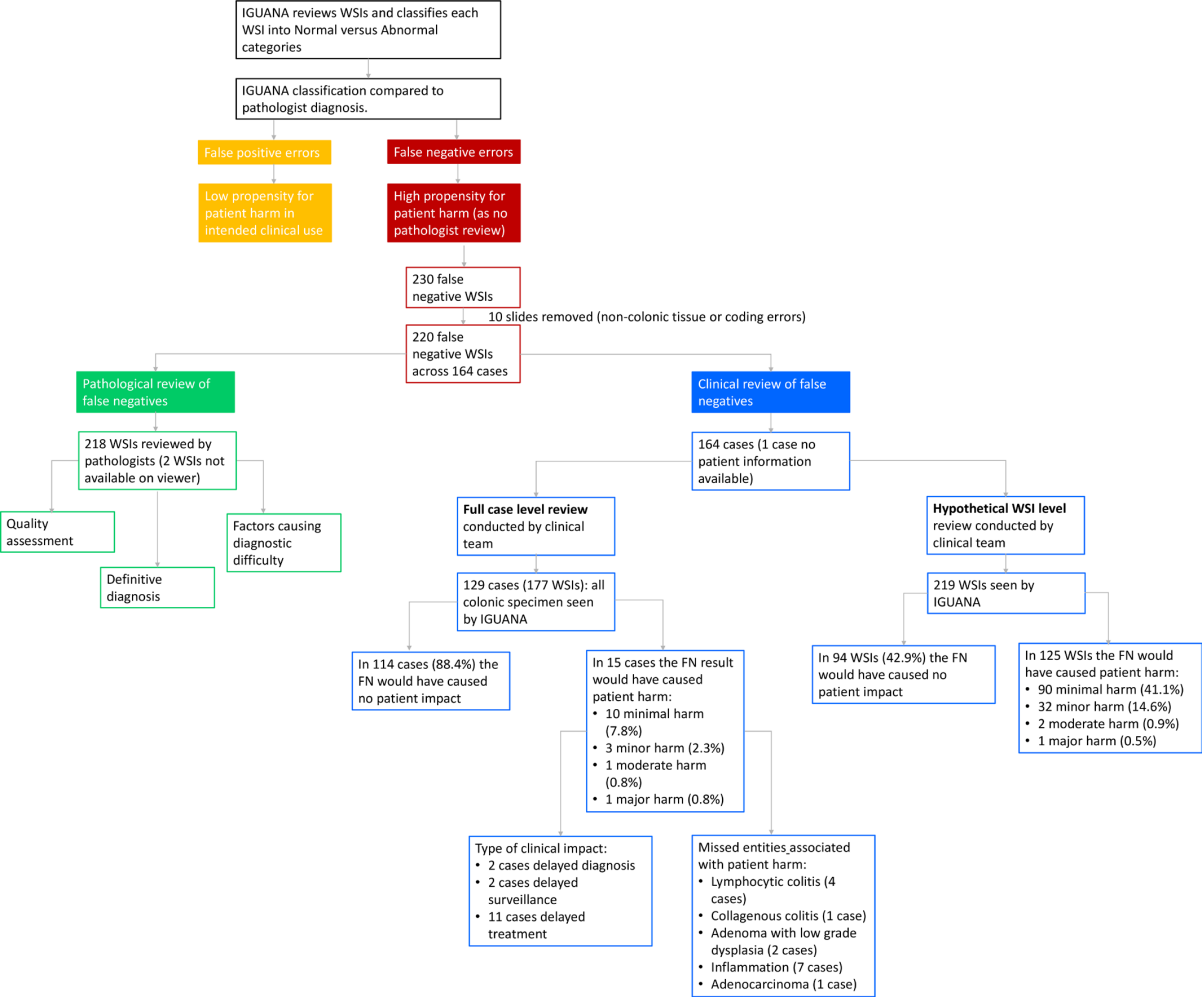
Overview of the review process. FN, false negative; IGUANA, Interpretable Gland-Graphs using a Neural Aggregator; WSI, whole slide image.

Figure 1 shows the review process that was conducted by the pathologists and clinical team.

### Pathological assessment

Two WSIs could not be retrieved on the image viewing software used for this research, leaving 218 WSIs for analysis.

#### Poor quality issues

There were only 6 of 218 WSIs that were felt to be poor quality by either pathologist. This suggests that poor quality issues were not a frequent problem hindering the interpretation of WSIs by IGUANA. The quality issues included the WSI being out of focus, folds in the tissue, or thick sections.

#### Entities missed by IGUANA

Table 1 shows precise diagnoses of the entities falsely classified as normal by IGUANA. This specificity is important because there can be heterogeneity in the features seen even within a single diagnosis. Although pathologists are aware of such feature diversity, this could contribute to IGUANA errors.

**Table 1:** Diagnoses of the 218 WSIs that were incorrectly classified as normal by IGUANA (ie, entities missed by the algorithm)

Entity	No. of WSIs
Acute/active inflammation	36
Acute/active inflammation (with granuloma)	10
Active chronic inflammation	22
Active chronic inflammation (with granuloma)	2
Chronic inflammation	32
Chronic inflammation (with granuloma)	4
Granuloma only	2
Eosinophilic inflammation	1
Ulceration	2
Spirochaetosis	3
Collagenous colitis	11
Lymphocytic colitis	10
Ischaemic colitis	2
Granulation tissue and fibrosis	1
Mild melanosis coli	1
Paneth cell metaplasia only	1
Haemorrhage and haemosiderin	1
Low-grade tubular adenoma	12
Low-grade tubulovillous adenoma	1
Hyperplastic polyp	20
Hyperplastic polyp (goblet cell rich variant/no or few serrations)	11
Possible polyp (low-grade tubular adenoma)— would do levels	8
Possible polyp (hyperplastic)—would do levels	7
Possible polyp (low-grade tubular adenoma and hyperplastic mixed)—would do levels	1
Leiomyoma	1
Lipoma	1
Atypical malignant cells in one fragment—possible contamination	2
Low-grade dysplasia in one fragment—possible contamination	2
Adenocarcinoma with signet ring cell morphology	1
Within normal histological limits (WNHL)/subtle abnormality pathologically insignificant (SAPI)	10

IGUANAInterpretable Gland-Graphs using a Neural AggregatorWSIwhole slide image

#### Reasons why a WSI might be difficult to interpret

The third component of the pathological review of the FN cases was to consider factors that might have made a WSI difficult to interpret and so contributed to the FN classification. Online supplemental table 1 documents the instances where comments were given as to why a case was difficult to diagnose correctly. The most common reason cited was that the WSI contained very focal pathological changes, so the features were only seen in a small region of the slide. Other factors included the slide containing mild inflammatory features and subtle/borderline features of a polyp.

### Clinical assessment

#### Patient level review

In the 129 cases (177 WSIs) where full case review was possible, it was found that in 88.4% of cases the FN(s) in the case would not have caused any patient harm. Where harm would have occurred (15 cases), this was predominantly labelled as minor harm, with only 1 case of major harm (table 2).

**Table 2:** Level of patient harm that would have occurred if the FN occurred in clinical practice, with a scoring system taken from the Royal College of Pathologists Guide to conduct a duty of care review^12^

Level of patient harm	Level of patient harm	Number of WSIs	Number of cases	Percentage of all 129 cases
1	No harm(no impact on care)	150	114	88.4
2	Minimal harm(no morbidity)	18	10	7.8
3	Minor harm(minor morbidity)	6	3	2.3
4	Moderate harm(moderate morbidity)	2	1	0.8
5	Major harm(major morbidity)	1	1	0.8

FNfalse negativeWSIwhole slide image

#### Reasons why FN diagnoses did not have any patient impact

The fact that for the vast majority of FN errors made by the algorithm, it was deemed that there would be no patient impact prompted consideration of the relevant protective factors limiting harm. These range from features relating to the type of pathology, the other investigations the patient had and symptom severity (table 3).

**Table 3:** Summary of the factors that meant an FN error did not lead to patient harm. (Some cases had more than one factor per case, resulting in a count of 117 across 114 cases)

Reasons for why an FN error did not result in patient harm	Number of instances
The features were detected in other biopsies from other colonic sites.	40
Low-risk polyp(s) removed and no further action needed.	39
The missed pathology was minor and no further action would have been taken had it been reported.	18
Focal active inflammation	7
Mild architectural changes/chronic inflammation	2
Granulomas	1
Mild and non-specific changes	2
Minor hyperplastic changes	1
Melanosis coli	1
Lipoma	1
Leiomyoma	1
Contamination while processing	2
The patient was already on the correct management pathway and no change was required.	10
The correct management pathway was commenced based on endoscopic findings.	3
The patient had minor symptoms and did not require any further management.	3
The symptoms self-resolved.	2
The patient was an asymptomatic patient having a check endoscopy/biopsy and no further treatment was required.	1
The correct management pathway was commenced based on other imaging.	1

FNfalse negative

#### Cases in which the FN would have caused some degree of patient harm

For the 15 cases where patient harm would have resulted from the FN, the way in which the FN would have impacted the patient’s management was categorised:

- Delayed diagnosis (2 cases)
- Delayed surveillance (2 cases)
- Delayed treatment (11 cases)

The conditions that were associated with these FN that caused harm were predominantly inflammatory diseases including active and/or chronic inflammation (n=6) and microscopic colitis (n=5), although also included a case of poorly differentiated signet ring adenocarcinoma and two instances of a tubular adenoma/tubulovillous adenoma with low-grade dysplasia (online supplemental table 2).

#### Review at WSI level

The hypothetical review at the WSI level considered, if this was the only slide that the patient had, and it had been missed, what would the impact have been? In this the rates of reported patient impact are much higher than the patient level assessment, with 57.1% of WSIs identified as hypothetically causing some level of patient harm (41.1% minimal harm, 14.6% minor harm, 0.9% moderate harm, 0.5% major harm) (online supplemental table 3). This presents an inflated impact of the FN errors and gives a less accurate understanding of the patient impact of errors, advocating for data collection to reflect (as closely as possible), the intended clinical scenario.

## Discussion

Current literature on AI tool evaluation in the healthcare setting fails to fully assess the impact of errors on patients. Although error rates are reported widely, how this translates into the clinical setting is rarely explored. There is no guidance on how to undertake a comprehensive, patient-focused error assessment for AI tools which likely hampers our understanding of the limitations of its use and thereby prevents widespread adoption. In this study, we have developed a systematic, patient-centred approach to the evaluation of an AI-based pathology tool, IGUANA, designed to screen between normal and abnormal colonic pathology slides.

### Pathological impact

The most significant missed diagnosis was a single WSI containing adenocarcinoma. This adenocarcinoma had a rarer morphology as it was predominantly composed of signet ring cells, in which the malignant cells contain a large mucin vacuole that pushes the nucleus to one side, giving a distinct appearance. This unusual morphology suggests a possible reason for why this was not detected.

Another area in which the algorithm struggled was in identifying hyperplastic polyps of the goblet cell rich variant, which have prominent goblet cells but few serrations (a more common defining feature of a hyperplastic polyp). For the detection of hyperplastic polyps IGUANA relied on the serrated architecture, with features such as gland lumen shape detected within the gland-graph network, however this is less prominent in goblet cell rich polyps.

This failure to detect certain subsets of entities based on their varying features is an example of hidden stratification that is known to occur with AI tools for medical imaging.^14^ This needs to be overcome by incorporating these rarer subtypes into training data sets.

**Figure 2:**
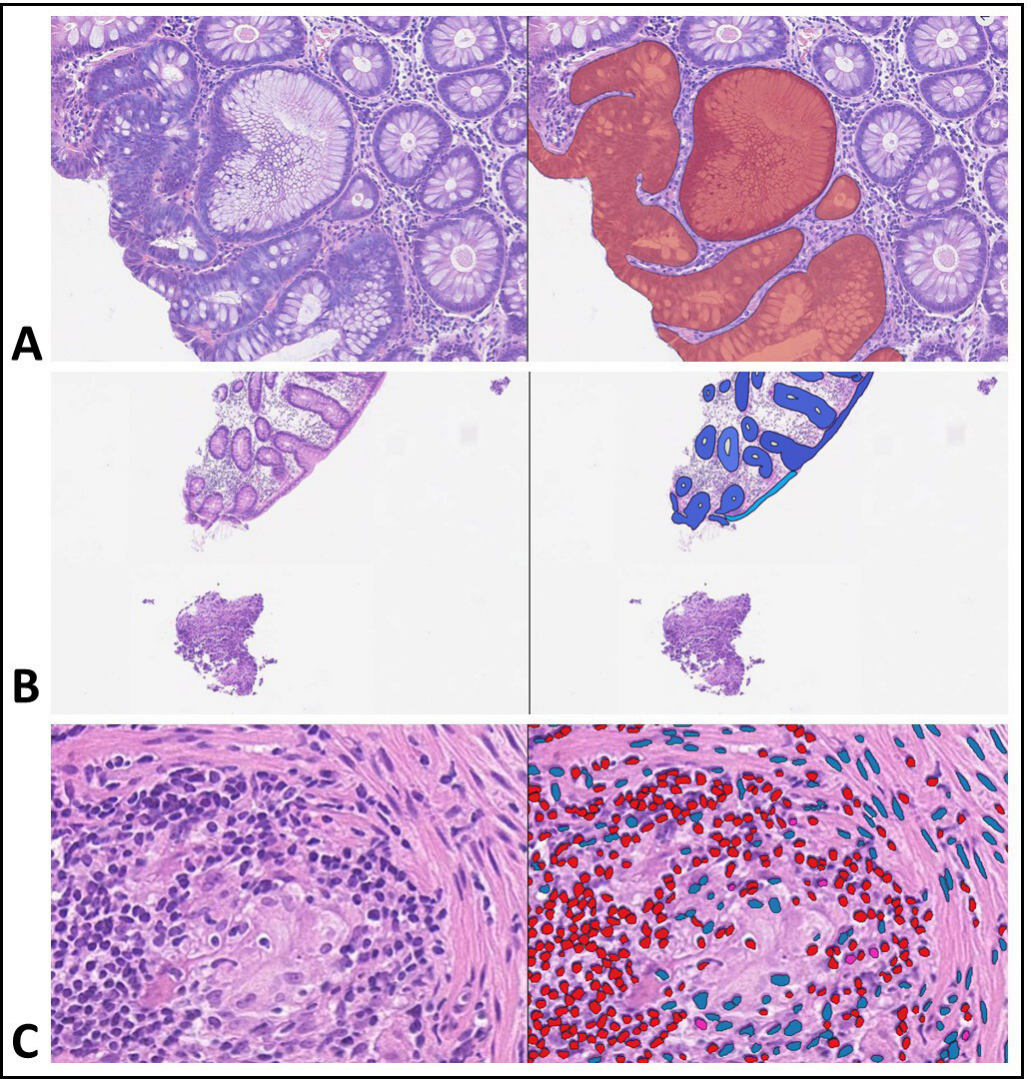
Example of slides where IGUANA failed to detect pathology. For each example the left image shows the histology and the right image shows the IGUANA overlay. (A) Low grade tubular adenoma. The small area of dysplasia was detected (shown as red gland overlay on right image), however as the rest of the slide was normal the aggregated result was that the slide was called normal. (B) Isolated fragment of dysplasia. This very small fragment was not detected by the algorithm at all, whereas the normal fragment above was correctly mapped (shown by blue gland overlay on right image). (C) Granuloma. IGUANA was not trained to detect granulomas. The overlay shows it classifying lymphocytes (red nuclei) and stromal cells (blue nuclei), including incorrectly classifying histiocytes as stromal cells. IGUANA, Interpretable Gland-Graphs using a Neural Aggregator.

There were several other reasons identified for why an FN error occurred, as shown in figure 2. In some slides, a focal area of abnormality was detected by IGUANA, but because the rest of the slide was normal the aggregated slide classification was an (incorrect) normal classification (figure 2 A). In other instances, the pathology was only present in a very small fragment of tissue, that was so small that it was not included in the gland model of the slide. This occurred with a fragment of dysplastic tissue in an otherwise normal slide and highlighted the need to include even small fragments in the slide graph network (figure 2 B).

In many cases IGUANA struggled to detect entities not included in the gland-graph model such as granulomas (figure 2 C), spirochaetosis and ulceration, highlighting the intricate correlation between training data and a successful AI tool. This knowledge shapes the further training requirements for a tool to improve its safety profile, tailoring the time-consuming task of algorithm training to pertinent disease areas. More surprisingly, several WSIs of acute and chronic inflammation were missed, despite comprising features that should be detectable by the algorithm. This warrants further investigation, which is complicated due to the complex nature of machine learning algorithms.

In some instances, IGUANA failed due to focal or mild pathology. In a similar way pathologists might occasionally struggle with such cases however, when a pathologist is confronted with subtle features they can take steps to facilitate making a diagnosis, such as a later re-review of a case, ordering more levels, immunohistochemistry or a second opinion. For example, with subtle features in a polyp, if the features are mild or unclear then further deeper levels could be cut to aid assessment, whereas an AI algorithm does not yet have such an ability. Several of the FN cases were felt to be possible polyps, meaning the pathologist in practice could perform further levels before making a diagnosis. Furthermore, a pathologist can interpret the findings in the context of the clinical and endoscopic information, which can aid in the correct classification of WSIs even with mild features. As IGUANA does not have the ability to perform further work or incorporate the endoscopic information, extra development is needed to cover these scenarios, in particular ensuring sensitive classification thresholds for the algorithm so any minor or uncertain cases are seen by a pathologist.

Some of the cases reviewed were felt to be within normal histological limits or only contained subtle abnormalities that were pathologically insignificant. There is no exact defined threshold at which a specimen goes from being normal to abnormal when subtle features are present and so the interpretation of such a slide varies between pathologists. This highlights the difficulty in turning the morphological spectrum of pathological features into a binary normal versus abnormal classification. This is likely to be an ongoing issue with future algorithm assessment and will contribute to error cases, however as these minor FN were not associated with any clinical implications, patient care is unlikely to be significantly impacted.

### Clinical impact

The majority of FN errors reported did not result in patient harm suggesting that even with a 4.4% WSI FN error rate, or 7.9% case-level FN error rate, this AI tool might be more suitable for adoption than this statistic portrays. It highlights that simply reporting error rates without addressing the clinical impact could lead to misrepresentation of AI tool safety. There are also several preventative scenarios in which the FN errors did not impact patient care, most commonly because the pathological features were detected in biopsies from other colonic sites, or because the entity was a low-risk polyp that was removed and then did not require any further action. Analysis of the impact of such errors in their clinical context is the only way to identify such preventative co-factors, with different factors relevant to other areas of AI tool use in pathology and across healthcare.

A key finding from this work was the importance of ensuring that the testing scenario is as close as possible to the true clinical scenario, even in early studies. In the IGUANA study there were several cases where only some of the endoscopic specimens were included. Although only using some specimens in a case is appropriate in terms of testing the algorithms’ ability to correctly classify slides, it limits the wider understanding of the tool, as demonstrated by a much lower rate of errors reported when a full case level, rather than hypothetical, assessment was performed. Studies should aim for case selection to be as representative as possible of the clinical scenario (which also helps to address other issues such as data generalisability).

Our study provides a comprehensive evaluation of how a tool would impact patients and current clinical practice. This is highly relevant to those working with the tool in practice including pathologists and clinicians, healthcare organisations considering implementing the tool and developers working to improve tool safety. This gives a greater understanding of a tool than statistical metrics and is likely to contribute to the increased transparency that is required to build trust in medical AI tools.^7^ It also facilitates targeted algorithm training based on missed pathological features and/or high-risk clinical scenarios.

However, despite these benefits there are several challenges. There is currently no framework for how developers should analyse error cases to promote patient safety. Although guidelines exist for the reporting of AI tools in healthcare, which mention the need to analyse error cases, there is no specification of how this is done.^15^,^17^ As such we had to build our own approach that was practical and feasible. This is time consuming, and not possible for all projects, but each study could identify the key clinical questions for their particular tool to address. In this work we focused on FN errors due to the greater propensity of these to cause patient harm than FP errors, and a similar pragmatic approach is required. As more tools move towards clinical implementation, it will be essential for guidelines and regulators to define requirements and guide developers on the necessary error assessment to meet safety requirements. Additionally, an approach like this requires a multidisciplinary team to understand both the tool and the clinical context, with collaboration between clinicians and computer scientists/tool developers being essential.

## Conclusion

AI tools require not only a detailed statistical analysis of the errors that are made but also an evaluation of how errors would impact patient safety and the potential implications for adoption into healthcare. A multidisciplinary team should be involved in healthcare AI tool generation and assessment so that the systems in which the AI tools will operate are understood. While developing an AI tool, it is not simply a matter of making it accurate and precise but also identifying protective factors inbuilt in the system that may protect against patient harm when the AI tool makes an error. In such a situation, a higher error rate may actually not lead to patient harm and could be deemed clinically acceptable. This paper highlights the importance, but also the challenges of a systems-based approach to the evaluation of AI tool safety and advocates for a similar approach to be more widely adopted.

## supplementary material

10.1136/bmjgast-2024-001649 online supplemental file 1

## Data Availability

Data are available upon reasonable request.
