# Supplementary material for: Evaluating the pathological and clinical implications of errors made by an artificial intelligence colon biopsy screening tool
Source: BMJ Open Gastroenterol. 2025 Jan 6;12(1):e001649. doi: 10.1136/bmjgast-2024-001649 (PMC11749196; doi:10.1136/bmjgast-2024-001649)
Supplement: online supplemental file 1 [file bmjgast-12-1-s001.docx]

Evaluating the pathological and clinical implications of errors made by an artificial intelligence colon biopsy screening tool.

Supplementary information

*Supplementary Table 1: Reasons documented as to why a WSI might be difficult to diagnose correctly. This table includes any instance that either pathologist mentioned a potential difficulty so duplicates exist across WSIs. Some WSIs had multiple comments made.*

| **Comment** | **Number of instances mentioned by a pathologist** |
| --- | --- |
| Mild inflammatory changes | 44 |
| Mild/subtle changes in a polyp | 35 |
| Borderline changes only (normal vs abnormal) | 13 |
| Rarer polyp subtype (gobet cell rich hyperplastic polyps with few serrations) | 14 |
| Focal changes | 64 |
| Mild/subtle changes microscopic colitis | 4 |
| Small micro-organism | 6 |
| Mild/subtle features of melanosis coli | 2 |
| Total | 182 |

*Supplementary table 2: The conditions where the missed diagnosis would have resulted in patient harm.*

| **Pathology type missed by IGUANA** | **Number of cases** |
| --- | --- |
| TA/TVA Low grade dysplasia | 2 |
| Active inflammation | 2 |
| Chronic inflammation | 1 |
| Active chronic inflammation | 1 |
| Inflammation with granulomas | 1 |
| Non-specific inflammation / architectural changes | 2 |
| Lymphocytic colitis | 4 |
| Collagenous colitis | 1 |
| Poorly differentiated signet ring adenocarcinoma | 1 |
| Total | 15 |

*Supplementary Table 3: Hypothetical harm level assessment done at a WSI level*

| **Hypothetical level of patient harm** | **Level of patient harm** | **Number of WSIs** | **% of 219 WSIs** |
| --- | --- | --- | --- |
| 1 | No harm (no impact on care) | 94 | 42.9 |
| 2 | Minimal harm (no morbidity) | 90 | 41.1 |
| 3 | Minor harm (minor morbidity) | 32 | 14.6 |
| 4 | Moderate harm (moderate morbidity) | 2 | 0.9 |
| 5 | Major harm (major morbidity) | 1 | 0.5 |
